# Biomolecular profiles of Arctic sea-ice diatoms highlight the role of under-ice light in cellular energy allocation

**DOI:** 10.1093/ismeco/ycad010

**Published:** 2024-01-10

**Authors:** Rebecca J Duncan, Daniel Nielsen, Janne E Søreide, Øystein Varpe, Mark J Tobin, Vanessa Pitusi, Philip Heraud, Katherina Petrou

**Affiliations:** School of Life Sciences, University of Technology Sydney, Sydney, New South Wales, 2007, Australia; Department of Arctic Biology, The University Centre in Svalbard, Longyearbyen, 9170, Norway; School of Life Sciences, University of Technology Sydney, Sydney, New South Wales, 2007, Australia; Department of Arctic Biology, The University Centre in Svalbard, Longyearbyen, 9170, Norway; Department of Biological Sciences, University of Bergen, Bergen, 5020, Norway; Norwegian Institute for Nature Research, Bergen, 5006, Norway; Australian Synchrotron—ANSTO, Clayton, Victoria, 3168, Australia; Department of Arctic Biology, The University Centre in Svalbard, Longyearbyen, 9170, Norway; Department of Arctic and Marine Biology, University in Tromsø (UiT), Tromsø, 9010, Norway; Centre for Biospectroscopy, School of Chemistry, Monash University, Clayton, Victoria, 3800, Australia; School of Life Sciences, University of Technology Sydney, Sydney, New South Wales, 2007, Australia

**Keywords:** sea-ice microalgae, Svalbard, single cell, lipid, fatty acid, under-ice light, inter-species variability

## Abstract

Arctic sea-ice diatoms fuel polar marine food webs as they emerge from winter darkness into spring. Through their photosynthetic activity they manufacture the nutrients and energy that underpin secondary production. Sea-ice diatom abundance and biomolecular composition vary in space and time. With climate change causing short-term extremes and long-term shifts in environmental conditions, understanding how and in what way diatoms adjust biomolecular stores with environmental perturbation is important to gain insight into future ecosystem energy production and nutrient transfer. Using synchrotron-based Fourier transform infrared microspectroscopy, we examined the biomolecular composition of five dominant sea-ice diatom taxa from landfast ice communities covering a range of under-ice light conditions during spring, in Svalbard, Norway. In all five taxa, we saw a doubling of lipid and fatty acid content when light transmitted to the ice–water interface was >5% but <15% (85%–95% attenuation through snow and ice). We determined a threshold around 15% light transmittance after which biomolecular synthesis plateaued, likely because of photoinhibitory effects, except for *Navicula* spp., which continued to accumulate lipids. Increasing under-ice light availability led to increased energy allocation towards carbohydrates, but this was secondary to lipid synthesis, whereas protein content remained stable. It is predicted that under-ice light availability will change in the Arctic, increasing because of sea-ice thinning and potentially decreasing with higher snowfall. Our findings show that the nutritional content of sea-ice diatoms is taxon-specific and linked to these changes, highlighting potential implications for future energy and nutrient supply for the polar marine food web.

## Introduction

The ecosystem within and directly below Arctic sea ice is highly seasonal, with light playing a critical role in its structure and functioning, through its influence on the productivity of the under-ice photosynthetic primary producers. The formation of landfast ice typically occurs in winter when darkness persists for 24 h. During this time, the early microbial community of sea-ice microalgae are captured within the brine pockets of the sea ice [1]. As the light returns to high latitudes in the early spring, dormant and vegetative sea-ice microalgae become active and begin to photosynthesise [2]. Early spring is characterized by sufficient inorganic nutrients supplied from the water below, but low light levels, which limit microalgal biomass and primary production [1, 3]. As spring progresses, light becomes abundant, resulting in peak productivity where ice algae can bloom, forming a key source of energy and nutrients for zooplankton and benthic reproductive cycles [4–6]. At our study sites at 78°N, the transition from perpetual darkness to constant daylight (the midnight sun) is rapid and occurs inside 42 days [7]. During this period, light reaching the sea-ice algae changes from being limiting to potentially harmful, if snow cover is absent [1, 8]. As summer approaches, despite abundant light, the lower nutrient concentrations [9, 10], increasing water temperatures and rapid brine drainage [11, 12], start to limit sea-ice algal productivity and, ultimately the higher summer temperature causes the ice to melt completely, releasing the microbial community living within the brine channels into the pelagic and benthic zones below [6].

The amount of light reaching the ice–water interface is dependent on snow depth and sea-ice thickness, which can be highly variable in space and time. As global temperatures rise with climate change, it is expected that snow and ice dynamics will change [13–15], and consequently the seasonal progression of the under-ice community will change as well. Whilst it is well-established that Arctic sea ice extent is declining [16–19] and becoming thinner [20–22], which alone would drive higher under-ice light levels, it is possible that the Arctic may experience increased precipitation in the form of higher snowfall in the short to medium term, because of increased storms and more open water [14, 23]. The influence of snow depth on light attenuation is far greater than sea ice alone. Snow-free ice can transmit up to 80% of incoming photosynthetically active radiation (PAR) [24, 25], whereas a 10 cm layer of fresh snow can effectively block light, reducing visible light transmission to <5% of incoming PAR [26]. As such, if the sea ice covered areas of the Arctic were to experience higher snowfall with global warming, despite thinner ice, under-ice light levels could be significantly reduced, modifying the growth conditions for the microalgae below.

Environmental conditions affect the allocation of photosynthetically derived carbon within polar ice algae, determining their biomolecular (i.e. lipid, carbohydrate, fatty acids, and protein) composition ([27], and references within). In turn, the biomolecular composition of ice algae determines the energy and nutrients available to the polar marine food web [28]. In actively growing ice algae, lipid may constitute up to 20% of dry weight and 60% of particulate organic matter composition [29, 30]. Lipids are the most energy-rich biomolecule with a caloric value of approximately twice that of carbohydrate and protein [31, 32] and transfer much of the energy between levels of the food web. Lipid content available at the primary production level is important for development, growth rate [33] and the amount of secondary production [34]. Because of the synthesis of fatty acids (FAs), in particular polyunsaturated fatty acids (PUFA), being tightly coupled to photosynthesis, eukaryotic algae are the main source of FAs to the marine food web [35, 36]. In sea-ice associated ecosystems, ice algae have been shown to be responsible for up to 50% of the FAs present in higher trophic levels, including fish, seals, and seabirds [37, 38]. In particular, FAs, including saturated (SAFA), monounsaturated (MUFA) fatty acids, and PUFAs, impact zooplankton fecundity and larval development [39, 40], as they are critical for membrane development, growth, and reproduction [35]. Their importance is evident from the high efficiency with which they are transferred through the trophic levels, with PUFAs shown to be transferred twice as efficiently from primary to secondary trophic levels compared with bulk carbon [41, 42]. Carbohydrates are also important biomolecules in terms of energy transfer [32], and play an important role in contributing to the cellular carbon pool [43]. Particularly under nutrient-depleted conditions, carbohydrates are a reserve product that can be drawn upon for lipid synthesis [44, 45]. Proteins are important for providing cellular nitrogen reserves and are the primary source of amino acids (AA) [46], which are vital for organism growth and survival, as regulators of metabolic pathways and as the structural elements of enzymes [47]. For heterotrophic organisms, non-essential AAs can be synthesized de novo; however, essential AAs must be provided by diet of which microalgae are the primary source in marine ecosystems [35]. Whilst polar ice algae typically have a relatively reduced photosynthate allocation to protein compared with other biomolecules [28, 48], they have a high transfer efficiency through food webs [49].

In the highly seasonal environment of the Arctic, in which productivity is severely restricted for much of the year, the provision of biomolecular energy from sea-ice algae is important for the transfer of energy through the polar marine ecosystem [50]. The value of sea-ice algae as food is linked also to the time in which they bloom. They are the primary source of carbon in the early spring [51–53] before pelagic phytoplankton proliferate [54], and some Arctic zooplankton have evolved to temporally align their reproductive cycle with this early food availability.

**Figure 1:**
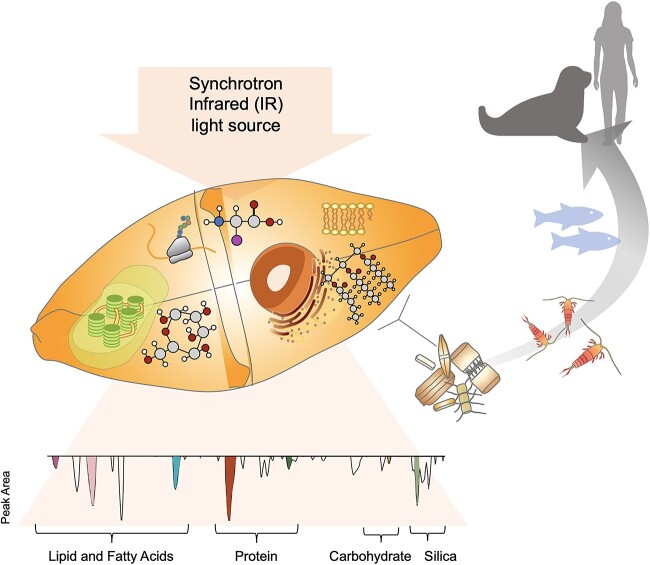
Conceptual model of the synchrotron IR light source measuring and the biomolecules within an individual cell, and the corresponding wavelengths on the IR spectrum (second derivative transformed) (bottom), in which the shaded sections represent the peak area of the biomolecules studied; a simplified overview of the transfer of these biomolecules up the polar marine food chain is displayed (right).

Studies on natural sea-ice algal communities have investigated nutrient content (e.g. lipid and protein content) at the community scale ([27], and references within), with only two studies looking at the effects of light on the biomolecular composition of Arctic diatoms at a taxon-specific level [55, 56]. Our study investigates the influence of the under-ice light conditions on the allocation of biomolecules, including lipids, proteins, and carbohydrates, in five Arctic ice-associated pennate diatoms: *Nitzschia frigida* (colonial), *Pleurosigma* spp. (solitary), *Navicula* spp. (solitary), *Haslea* spp. (solitary), and *Entomoneis* spp. (solitary), taken from natural communities within the landfast sea ice in Svalbard, Norway. Using synchrotron-based Fourier transform infrared (s-FTIR) microspectroscopy (Fig. 1) to analyse individual cells, we uncover taxon-specific patterns in biomolecular production and allocation, providing insight into how sea-ice algae nutritional content may change with climate-driven shifts in community composition.

## Materials and methods

### Study area

**Figure 2:**
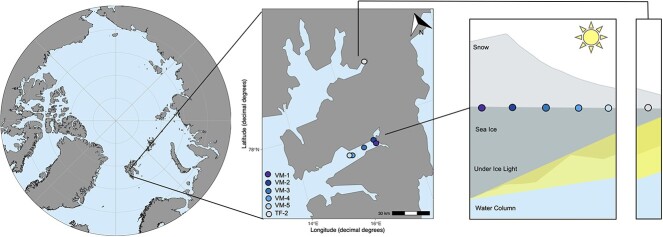
Location of Svalbard, Norway, within the Arctic (left), sampling locations visited between April and May 2021 in Svalbard (middle), overview of the snow depth, sea ice thickness, and under-ice light at each of the sampling stations (right).

This study was conducted within Tempelfjorden and Van Mijenfjorden in Svalbard, Norway (Fig. 2; Table S1) on 30 April and 4–5 May 2021, respectively. Within Van Mijenfjorden, five sites were sampled once along a transect from the inner to the outer fjord (VM-1–5), capturing a gradient of ice thickness and snow depth. A site from Tempelfjorden (TF-2) was sampled once and included because of its thinner sea ice and snow depth, providing data from an environment of higher light transmissivity to the ice–water interface. Both fjords are located on the west coast of Svalbard and are influenced by glacial run-off [57]. Additionally, both fjords are comprised of an outer basin up to 120-m deep and inner basin up to 70-m deep (Van Mijenfjorden) and 60-m deep (Tempelfjorden). However, unlike Tempelfjorden, Van Mijenfjorden is a partially enclosed fjord meaning it has longer and more predictable sea ice cover [57]. Detailed analysis of the under-ice protist community composition at all sites is available in Duncan *et al*. [58].

### Sample collection

At each sampling site, six ice cores were extracted ~0.5–1-m apart, using a Kovacs core barrel (9 cm diameter; Kovacs Enterprise, Oregon, USA). The bottom 3 cm (at the ice–water interface) of each core was retained, as this is where the microbial community was concentrated [59–61]. Cores were then pooled into triplicates, as cores 1–2, 3–4, and 5–6, and 100 ml of filtered sea water (GF/F, nominal pore size 0.7 μm) was added for every centimetre of core to minimize osmotic stress [62, 63] after which the samples were allowed to melt in darkness for 24 h at 4°C. To concentrate the cells, 100 ml from each of the three samples was centrifuged at 1000 rpm (Universal 320, Hettich, Germany) for 4 min and the supernatant removed. The remaining sample was then transferred to 2 ml Eppendorf tubes and centrifuged at 1000 rpm (Mikro 185, Hettich) for 2 min before the supernatant was removed, and the sample was fixed by addition of formalin (5% v/v) in FSW for later analysis.

### Environmental parameters

#### Physical parameters

Three snow depth measurements were taken to the nearest 0.5 cm using a standard ruler, to determine the average snow depth per core. Ice thickness was measured using a Kovacs ice thickness gauge. Water temperature was measured just below the ice–water interface using a CTD probe (STD/CTD SD204, SAIV A/S: Bergen, Norway). At each sampling site, ~100 ml of the water from directly below the ice surface was collected in acid washed bottles for nutrient analysis. The samples were frozen until analysis, when they were melted and 4 M H_2_SO_4_ was added for preservation in transport for analysis at Akvaplan-niva, Norway. The nitrate plus nitrite (NO_3_^−^ + NO_2_^−^) (NOx), phosphate (PO_4_^3−^), silicic acid (Si(OH)_4_), and ammonium (NH_4_) concentrations (μM) were measured simultaneously on a San++ 5000 automated analyser (Skalar: Breda, the Netherlands), with separate analysis channels for the four nutrients. The detection limits were 0.02 μM for NOx, 0.01 μM for phosphate, 0.25 μM for silicic acid, and 0.3 μM for ammonium. Stable isotope analysis of the 0–3 cm section of sea ice was performed in an elemental analyser isotope ratio mass spectrometry system, as previously published [64].

#### Light measurements and modelling

Incoming photosynthetically active radiation (PAR) was measured at each sampling site in Van Mijenfjorden using a LI-190 quantum air sensor placed on the sea ice surface and a LI-192 underwater quantum sensor placed on a weighted frame positioned through a 10-cm hole in the sea ice, with measurements collected using a LI-1500 Data Logger (LI-COR, Nebraska, USA). To avoid shadowing of the measurement area, all sensors faced south with operations performed north, and the area was undisturbed. However, at Tempelfjorden, the underwater quantum sensor failed. Therefore, to ensure light transmittance values were available from all sampled sites and determined using a consistent methodology, and to utilize our unique *in situ* surface light measurements, the under-ice light measurements were modelled. Light at the ice–water interface under the sea ice was estimated using *in situ* measured irradiance at the top of snow and ice, and then attenuation through snow and ice was determined using attenuation coefficients of 20 m^−1^ for snow, 5 m^−1^ for the top 10 cm of ice, and 1 m^−1^ for ice below the top 10 cm [65, 66], using the following equation:


(1) \begin{equation*} \mathrm{E_Z}=\mathrm{E}_0\cdot \exp\ \left(-\mathrm{K_d} \cdot \mathrm{Z}\right) \end{equation*}

where (E_Z_) is irradiance at sampling depth, E_0_ is the surface irradiance (μmol photons m^−2^ s^−1^), K_d_ is the diffuse light attenuation coefficient (m^−1^), and Z is the sampling depth (m). Light values at the ice–water interface were converted to percent incoming PAR to account for the measured *in situ* irradiance above-ice being taken at various times of day and with a range of cloud coverage conditions, which have a substantial effect on light levels [67, 68]. Below we use light transmissivity as a descriptive term for percent incoming PAR and have divided the sites into those receiving < 5% incoming PAR as low light transmissivity (LLT) sites, and those receiving > 5% incoming PAR as high light transmissivity (HLT) sites.

#### Species-specific biomolecular composition by Fourier transform infrared

**Figure 3:**
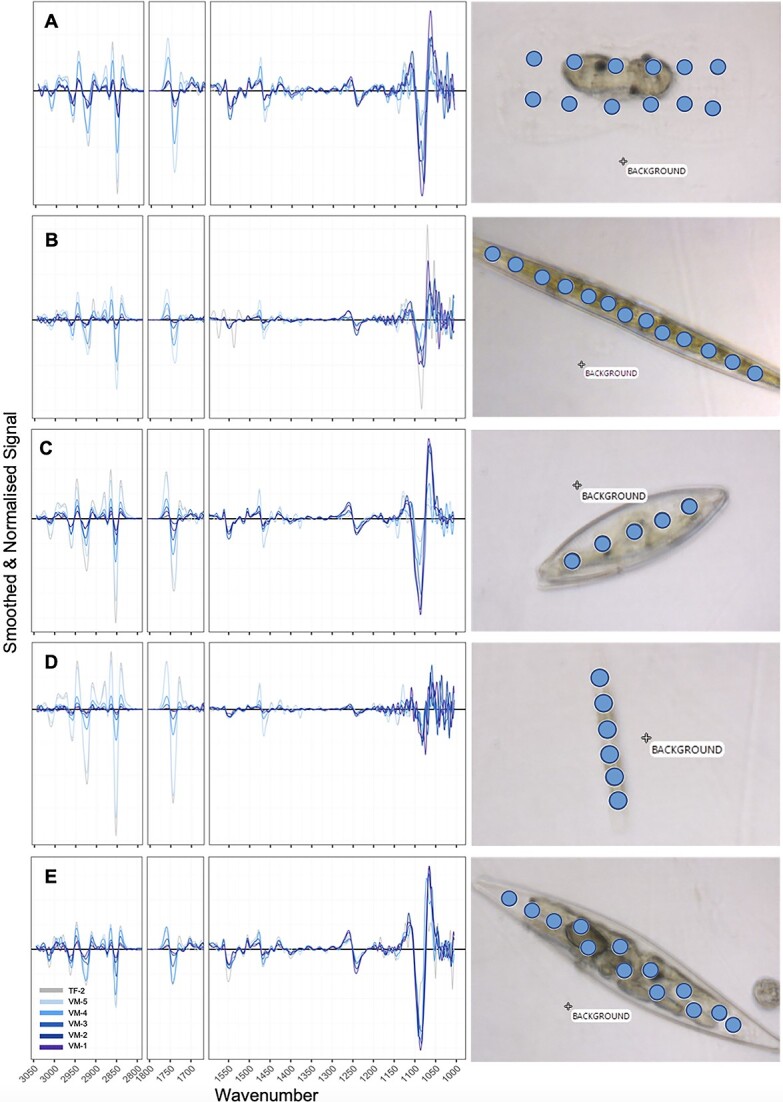
Smoothed and normalized spectra of each of the five taxa, (A) *Entomoneis* spp., (B) *Haslea* spp., (C) *Navicula* spp., (D) *N. Frigida,* (E) *Pleurosigma* spp., with each site denoted through a gradient; images of example cells of each taxa (right) with dots denoting the s-FTIR measurement points (where the aperture (actual measuring area) for each point was larger than the point indicated), demonstrating the entire cell contents were measured.

The biomolecular composition of five selected taxa (*N. frigida*, *Pleurosigma* spp., *Navicula* spp., *Haslea* spp., and *Entomoneis* spp.) (Table S2) was determined using synchrotron-based FTIR microspectroscopy on hydrated, formalin-fixed (5% v/v final concentration) cells. All cells were measured as single cells, i.e. not dividing or associated with a chain. The *Navicula* spp. group consisted primarily of *Navicula transitas, Navicula directa,* and *Navicula valida*. Samples were loaded (3 μl) directly into a micro-compression cell between two 13 mm diameter 0.5-mm thick CaF_2_ windows [69]. Using the Infrared (IR) Microspectroscopy Beamline at the Australian Synchrotron, Victoria, spectral data of individual cells (between 1 and 20 cells per taxon per site, Table S3) were collected in transmission mode. Each biomolecule absorbs a specific range of IR wavelengths, and a set of well-defined absorbance bands between 3050–2800 cm^−1^, and 1770–1100 cm^−1^ have been determined (Table 1). Spectra were acquired over the measurement range 4000–800 cm^−1^ with a Vertex 80v FTIR spectrometer (Bruker Optic, Ettlingen, Germany) in conjunction with an IR microscope (Hyperion 3000, Bruker) fitted with a narrow-band mercury cadmium telluride detector cooled with liquid nitrogen. The use of hydrated cells as opposed to desiccated samples has been shown to limit light scattering effects [70]. Co-added interferograms (sample *n* = 32, background *n* = 64) were collected at a wavenumber resolution of 4 cm^−1^. To allow for measurements of individual cells, all measurements were made in transmission mode, using a measuring aperture diameter of 6.9 μm (area = 37.4 μm^2^) for the smaller taxa (*N. frigida*, *Navicula* spp., and *Haslea* spp.) and 12.5 μm (area = 122.7 μm^2^) for the larger taxa (*Pleurosigma* spp*.* and *Entomoneis* spp.). All cells were measured with multiple points across the cell surface to account for heterogeneity in the cell structure and distribution of biomolecules (Fig. 3). Spectral acquisition and instrument control were achieved using Opus 7.5 software (Bruker). Analyses were performed within 6 months of samples being collected and fixed. All samples were kept refrigerated between fixation and analysis.

**Table 1:** IR band assignments for s-FTIR microspectroscopy used in this study.

**Wave number (cm** ^**−1**^**)**	**Band assignment**	**Reference**
~3011	ν(C–H) of cis C═CH– from unsaturated lipids	Vongsvivut *et al*. [108]
~2960	ν_as_(C–H) from methyl (–CH3) groups of lipids and proteins	Vongsvivut *et al*. [108]
~2921	_νas_(C−H) from methylene (−CH_2_) from saturated lipids	Vongsvivut *et al*. [108]
~2852	_νs_(C−H) from methylene (−CH_2_) from saturated lipids	Vongsvivut *et al*. [108]
~1744	_ν_ (C = O) from ester carbonyl group from lipid triglycerides and fatty acids	Vongsvivut *et al*. [108]
~1549	Amide II mode from proteins; mainly δ(N−H) of amides	Heraud *et al*. [109]
~1400	_νs_(COO^−^) from carboxylated molecules	Sackett *et al*. [110]
~1377	δ_s_(CH_3_) and δ_s_ (CH_2_) of lipids and proteins	Heraud *et al*. [111]
~1241	_νas_(PO_2_^−^) of the phosphodiester backbone of nucleic acids, phosphorylated proteins, and phosphorylated lipids	Whelan *et al*. [112] and Sackett *et al*. [110]
~1146	_νs_(C−O) from carbohydrates	Heraud *et al*. [113]
~1080	_νs_(Si−O) from silica	Beardall *et al*. [114] and Sackett *et al*. [115]

#### Data analyses

IR spectral data were analysed in R v4.2.2 [71]. Data were smoothed (4 pts either side) and second derivative (third-order polynomial) transformed using the Savitzky–Golay algorithm from the prospectr package [72] and then normalized using Standard Normal Variate (mean centred and SD of 1) (Fig. 3). Biomolecular content for each measured cell was estimated based on integrating the area under each assigned peak (Table 1), using the Beer–Lambert Law, which assumes a direct relationship between absorbance and analyte concentration to determine metabolite content [73].

Relationships between biomolecular content and environmental variables (% incoming PAR, nitrate and silicate concentration in the water at ice–water interface, bottom-ice temperature and salinity and water temperature at the ice–water interface) were investigated using Spearman’s rank correlation coefficient (Table S4). As % incoming PAR was the most highly correlated with the biomolecular profile, relationships between biomolecular content and percent incoming PAR were estimated using principal component analyses (PCA) and with linear regressions applied to the mean peak area at each incoming % PAR level (i.e. each sampling site) (± 95% confidence interval) for each taxon. Because of the difference in absorption properties of biomolecules, the integrated peak areas provide relative changes between samples, meaning any quantitative measure of change can only be applied within compounds. The Shapiro–Wilks [74] test for normality showed the data required log_10_ transformation before analysis. The number of cells measured ranged from 1 to 20 per taxa, per site (Table S3). Because of the low abundance of *Haslea* spp. and *Pleurosigma* spp. at TF-2, however, no confidence interval was applied to the linear regressions beyond 15.2% incoming PAR. Relationships between lipid and protein content, lipid and carbohydrate content, and carbohydrate and protein content, with increasing light reaching the ice–water interface, were also investigated using linear regression. Fixed factor linear regression models, with under-ice light level (HLT vs LLT) as the factor, were used to determine that these regressions were improved when separated according to HLT sites (VM-4, VM-5, TF-2) and LLT sites (VM-1–3) sites. Statistical significance of the regressions was concluded based on the *F* statistic (*P* < .05) and strength of fit estimated using *R*^2^. The residuals of all regressions were verified for homoscedasticity. All analyses were performed using R Studio v. 2022.02.03 [71] and the add-on packages ggplot v. 3.3.6 [75], dplyr v. 1.0.8 [76], corrplot [77], and vegan v. 2.6–4 [78].

## Results

### Physical parameters

Within Van Mijenfjorden, snow depth and ice thickness decreased towards the fjord opening. The outermost site (VM-5) had ice thickness of 52 ± 3.5 cm and a snow depth of 4.8 ± 3.5 cm, allowing 15% incoming PAR at the ice–water interface, and the innermost site (VM-1) had ice thickness of 92 ± 3.2 cm and snow depth of 14.3 ± 0.7, allowing 1.5% incoming PAR (for data on all sites, see Table 2). The Tempelfjorden site (TF-2) had the highest incoming PAR at 23%, with an ice thickness of 38 ± 1.1 cm and snow depth of 3.4 ± 0.7 cm. Based on the average incoming PAR of 640 μmol m^−2^ s^−1^, measured *in situ* at the snow surface of the sampling sites, this equates to a range of 8–148 μmol m^−2^ s^−1^ transmitted through the snow and ice to the bottom ice community. In mid-April, all VM sites experienced < 3% incoming PAR; however, by late April, VM-4 and VM-5 experienced 23% and 14% incoming PAR, respectively. Seawater nutrient concentrations were relatively high, with nitrate concentrations ranging from 2.18 μM (VM-4) to 1.92 μM (TF-2) (Table 2) and silicate concentrations ranging from 2.81 μM (VM-4 and 5) to 2.5 μM (VM-1). Taken from the sea ice, stable isotope of carbon (δ^13^C_VPDB_ (‰) was more enriched at HLT sites (*t*(2) = 4.02, *P* < 0.05), averaging −17.42 ± 3.2 at the HLT sites (TF-2, VM-4, VM-5) and − 24.85 ± 0.5 at the LLT sites (VM-1–3). Ice temperature in the 0–3 cm section was between −2.1 and −2.2°C at all VM sites and −2.7 at TF-2, whereas the under-ice water temperature ranged from at −1.61°C (VM-4) to −1.86 (VM-2). Bottom bulk ice salinity ranged from 10.7 (VM-2) to 3.7 (VM-4). Given that bottom ice temperature was consistently below the seawater freezing point (−1.7°C), brine volume remained well within the reasonable range for communities to inhabit sea ice (>5%) [77] and all sites were nutrient replete (>1.9 μM), these environmental variables were unlikely significant drivers for any observed metabolomic changes. In contrast, under-ice light transmittance (through snow and ice), was the most variable environmental variable across sites, but also the one that correlated most strongly and consistently with biomolecular content (Table S5) and therefore the focus of this study. For more details and further physical parameters, see Table 2 and [58].

**Table 2:** Parameters measured associated with sea ice core extraction; snow depth (± SD, *n* = 18), ice thickness (± SD, *n* = 6), % incoming PAR, and under ice light (μmol m^−2^ s^−1^); measurements from within the bottom 3 cm of sea ice core: temperature (°C), bulk salinity (ppt), brine salinity (ppt), brine volume (% of ice volume), chlorophyll *a* concentration *(*mg/m^2^) (*n* = 3), particulate organic carbon to particulate organic nitrogen ratio (C:N); parameters measured in under-ice water at each sampling site: ammonium (NH_4_), silicate (Si(OH)_4_), nitrate (NO_3_), and phosphate (PO_4_) concentrations (μM) and temperature (°C), where N.D. denotes not measured.

Sea ice												Under-ice water				
Date	Station	Snow depth (cm)	Ice thickness (cm)	% incoming PAR	Light (μmol m^−2^ s^-1)^	Ice temperature (°C)	Ice salinity (ppt)	Brine salinity (ppt)	Brine volume (%)	Chlorophyll (mg/m^2^)	C:N	NH_4_ (μM)	Si(OH)_4_ (μM)	NO_3_ (μM)	PO_4_ (μM)	Temperature (°C)
5.5.21	VM-1	14.3 ± 0.7	92 ± 3.2	1.5	12.52	−2.1	6.5	37.36	15.57	0.48 ± 0.3	6.09	<1	2.50	2.10	0.20	−1.71
5.5.21	VM-2	15.8 ± 2.1	78 ± 3.0	1.3	8.66	−2.2	10.7	39.07	24.49	0.29 ± 0.0	5.73	<1	2.60	2.08	0.20	−1.86
4.5.21	VM-3	10.0 ± 0.9	74 ± 2.6	4.2	17.14	−2.2	10.4	39.07	23.81	1.90 ± 0.2	6.02	<1	2.71	2.14	0.21	−1.81
4.5.21	VM-4	7.0 ± 3.2	50 ± 2.2	10.3	68.17	−2.1	3.7	37.36	8.86	0.63 ± 0.1	7.50	<1	2.81	2.18	0.23	−1.61
4.5.21	VM-5	4.8 ± 3.5	52 ± 3.5	15.2	99.29	−2.2	5.6	39.07	12.82	0.86 ± 0.2	6.92	<1	2.81	2.14	0.23	N.D.
30.4.21	TF-2	3.4 ± 0.7	38 ± 1.1	23.2	104.19	−2.7	9.2	47.53	17.25	2.82 ± 1.0	10.18	<1	2.71	1.92	0.20	−1.80

### Species-specific biomolecular composition

**Figure 4:**
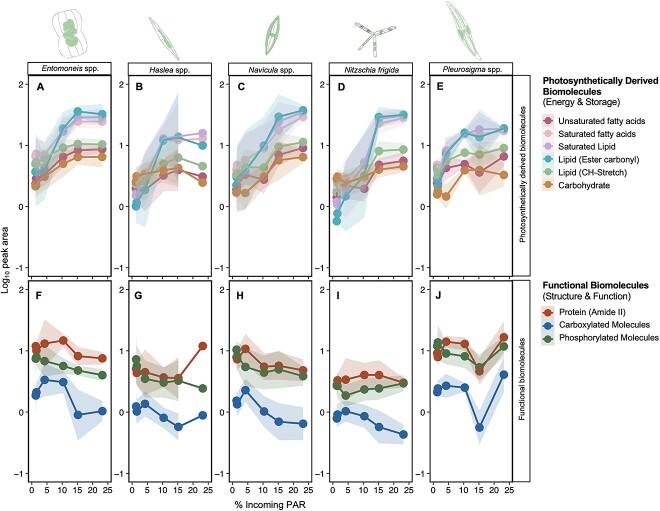
Mean cell-specific biomolecular content (based on normalized peak areas—see Table 1) for photosynthetically derived (energy-rich, storage) biomolecules (unsaturated fatty acids, saturated fatty acids, saturated lipids, lipids (ester carbonyl), lipids (CH-stretch II), and carbohydrates) (A) *Entomoneis* spp., (B) *Haslea* spp., (C) *Navicula* spp., (D) *N. Frigida,* (E) *Pleurosigma* spp., and functional (structural and cell function) biomolecules (protein (amide II), carboxylated molecules, phosphorylated molecules) (F) *Entomoneis* spp., (G) *Haslea* spp., (H) *Navicula* spp., (I) *N. Frigida,* (J) *Pleurosigma* spp. as a function of the proportion of light reaching the ice–water interface; shading indicates 95% confidence intervals, applied to log-transformed data.

Across all five taxa, lipid (ester carbonyl) and carbohydrate content generally increased with increasing percent incoming PAR until 15% surface irradiance, after which, the content plateaued or declined in all taxa except for *Navicula* spp., which saw a continued increase up to 23% incoming PAR (Fig. 4; Table S5). The other photosynthetically derived biomolecules, including unsaturated FAs, SAFAs, saturated lipids, and lipids (CH-stretch II), followed the same increasing trend with percent incoming PAR until ~15% (Fig. 4; Table S5). We saw no clear trend associated with percent incoming PAR for the other functional biomolecules, i.e. protein (amide II) and phosphorylated molecules (Fig. 4; Table S5). Carboxylated molecules experienced a decline with increasing percent incoming PAR in all taxa except *Pleurosigma* spp. (Fig. 4).

**Figure 5:**
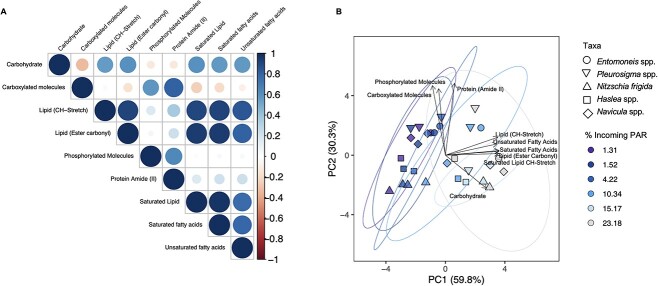
(A) Correlation plot based on Spearman’s rank correlation coefficient for biomolecular content across all taxa and sampling sites, where gradient is used to represent the strength of correlation (and blue represents positive whilst red represents negative correlation); (B) PCA of biomolecular content at each under-ice light level for all taxa combined (*Entomoneis* spp., *Haslea* spp., *Navicula* spp., *N. Frigida,* and *Pleurosigma* spp.); direction and strength of individual biomolecules are displayed with ordination bi-plot overlay.

The correlation matrix shows clear separation in the relationships between photosynthetically derived and structural biomolecules (Fig. 5A). We saw strong positive correlations (>0.75) amongst the lipid compounds (ester carbonyls, saturated lipids, SAFAs, and unsaturated FAs), and moderate positive correlations with carbohydrates (>0.50, Fig. 5A). Furthermore, these photosynthetically derived biomolecules were only weakly correlated with protein and phosphorylated molecule content (<0.3), and negatively correlated with carboxylated molecules (<−0.3). Structural biomolecules (protein, phosphorylated molecules, carboxylated molecules) were positively correlated with one another (>0.6, Fig. 5A).

Analysing all peak areas of all five taxa from all sites, we found clustering according to a light gradient (Fig. 5A), with PC1 explaining 61.9% of the variation in biomolecular content, correlating with the gradient in percent incoming PAR. The difference observed across PC1 was driven by lipid, saturated lipid, SAFA, unsaturated FA, lipid (CH-stretch II), and carbohydrate bands, corresponding with their measured increase with increasing transmitted irradiance (Fig. 4). The next main source of variation along the PC-2 was driven by the difference in protein (amide II), carboxylated molecules, and phosphorylated molecule content, explaining 28% of the variation (Fig. 5B). Separation of the data by taxa reveals that this variation is likely species derived, with *N. frigida* and *Haslea* spp., clustering separately to *Navicula* spp., *Pleurosigma* spp., and *Entomoneis* spp., particularly within the LLT sites (Fig. 5B).

**Figure 6:**
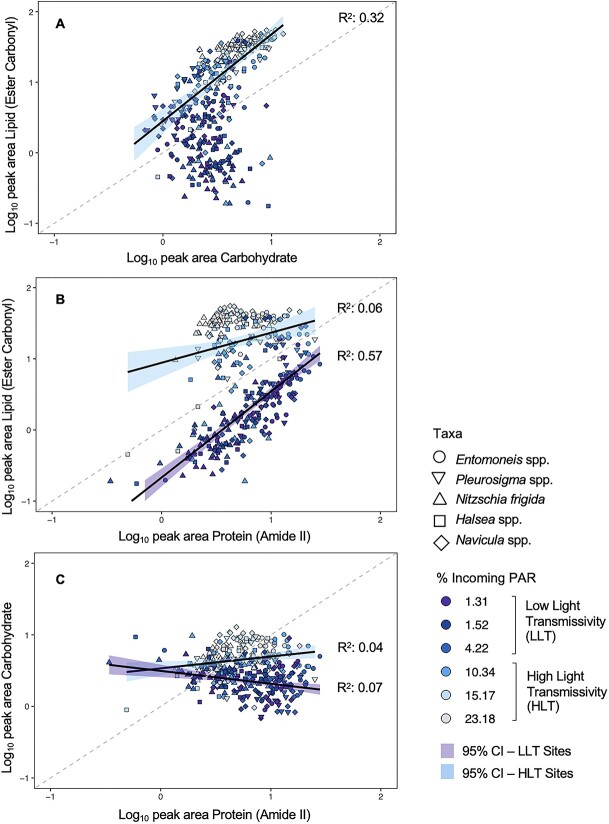
(A) Lipid (ester carbonyl) vs. carbohydrate content, (B) lipid (ester carbonyl) vs. protein (amide II) content, and (C) carbohydrate vs protein content, based on normalized peak areas, for all taxa combined and. Data are divided into HLT sites (VM-4, VM-5, TF-2) and LLT sites (VM-1–3), with light levels indicated with a gradient fill and taxa are denoted by shape; the data are fitted with linear regressions, with 95% confidence intervals (shading), applied to log-transformed data; only statistically significant regressions are shown.

To estimate the key changes to biomolecular content and carbon allocation in the cell, the relationships between lipid, protein, and carbohydrate were investigated for each taxon using linear models. Including low (<5% incoming PAR) and high (>5% incoming PAR) light as fixed factors improved the model outcome, resulting in two regressions for each comparison. A positive correlation between lipid and carbohydrate was observed at HLT conditions only (*F*_1,182_ = 92.95, *P* < .05; *R*^2^ = 0.34, Fig 6A) and the same correlation was observed for each species individually (Table S5). No correlation between lipid and carbohydrate was observed at LLT sites when all species were considered together (Fig. 6A). We saw a positive correlation between lipid and protein in all species under LLT conditions (*F*_1,98_ = 271.8, *P* < .05; *R*^2^ = .58, Fig 6B; Table S5). This was also observed at HLT when all species were considered together (*F*_1,182_ = 10.15, *P* < .05; *R*^2^ = 0.06, Fig. 6B), driven by the positive correlations in *Entomoneis* spp. and *Haslea* spp. only (Table S5). A strong parallel shift in increasing lipid content from LLT to HLT conditions was observed in all five taxa (Fig. S1). For carbohydrate, a weak negative correlation relationship with protein content was observed in the LLT sites when all species were considered together (Fig 6C), whereas a positive correlation was observed at HLT sites. Similar to lipids, *Entomoneis* spp. and *Haslea* spp. only exhibited increases in carbohydrate content under HLT, with minimal changes to protein content (Table S5).

**Figure 7:**
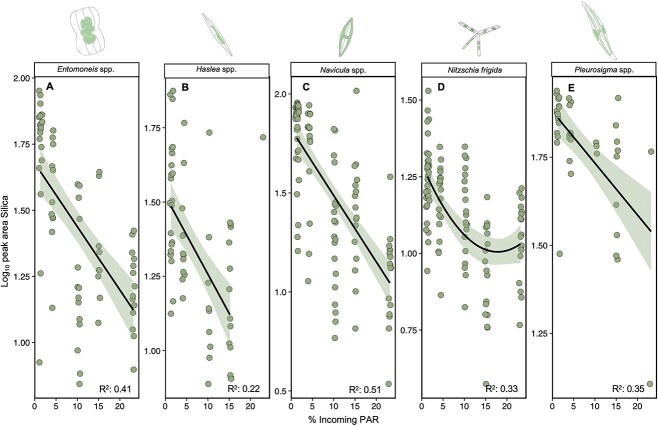
Silica content (based on normalized peak areas) with increasing percent photosynthetic active radiation (PAR) reaching the ice–water interface, per taxa; each data point represents one measured cell; the data are fitted with linear regressions, with 95% confidence intervals (shading), applied to log-transformed data; only statistically significant regressions are shown.

Integration of the silica peaks (maxima at 1080 cm^−1^) for each species and site revealed that silica concentration declined with declining percent incoming PAR in all species (Fig. 7A–E). For *Entomoneis* spp. (*F*_1,66_ = 44.94, *P* < .05; *R*^2^ = 0.41), *Haslea* spp., (*F*_1,55_ = 14.77, *P* < .05; *R*^2^ = 0.22), *Navicula* spp. (*F*_1,104_ = 107.6, *P* < .05; *R*^2^ = 0.51), and *Pleurosigma* spp. (*F*_1,40_ = 21.67, *P* < .05; *R*^2^ = 0.35), the decline in silica content was linear with declining percent incoming PAR, whereas for *N. frigida* (*F*_2,106_ = 26.2, *P* < .05; *R*^2^ = 0.33), the decline was observed until ~15% incoming PAR, after which it plateaued, suggesting a minimum level of silicification had been reached.

## Discussion

Biomolecules supplied by sea-ice microalgae to the polar marine food web are important, particularly as the primary food supply early in the season, but also as a source of essential fatty acids for zooplankton reproduction. Microalgal biomolecular composition, however, is environmentally determined [27] and therefore even subtle shifts in the physio-chemical conditions of the surrounding environment can affect the nutritional content supplied to the marine food web. In this study, key environmental variables (temperature, nutrients, salinity, etc.) varied minimally across the sites and were generally within the expected range for spring [79], whereas snow and sea-ice thickness varied more and the resulting under-ice light transmittance strongly correlated with biomolecular changes.

Across all five taxa, we found that increasing under-ice light availability led to an increase in lipids, fatty acids, and carbohydrates, and that lipid accumulation was preferentially allocated over carbohydrates at higher irradiances. In most cases, the increases in lipids, fatty acids, and carbohydrates were observed until ~15% incoming PAR, at which point saturation occurred. This observed threshold could be because of the highest irradiances causing photoinhibition and thus limiting further biomolecular production [80–82], as the measured irradiances for VM-5 and TF-2 were outside the typical spring bloom range (>20% incoming PAR or >100 μmol m^−2^ s^−1^) [27]. Evidence of photoinhibition has been observed in sea-ice microalgae at similar light levels [82–84] and is generally a result of synthesis of photoprotective pigments, changes to chlorophyll content, and downregulation of photosystem II [84]. The greatest increase in lipid content with increasing under-ice light was observed in *N. frigida* until 15% incoming PAR, which aligns with previous observations that *N. frigida* accumulated higher lipid stores than *Attheya* spp. and pennate ribbon colonies [56]. The exception to the observed light threshold was *Navicula* spp., which continued to increase lipid and carbohydrate content beyond 15% incoming PAR, suggesting morphological or photophysiological differences in this taxon. One explanation for this difference could be that *Navicula* spp. often have large central regions with chloroplasts concentrated along the cell wall [85] and low intracellular chlorophyll under high light conditions as a photoadaptive strategy [86], minimizing photoinhibition and leaving substantial space for lipid accumulation in oil droplets within the cytoplasm [87]. In Antarctic sea-ice microalgal communities, *Navicula* spp. were found also to have a higher lipid content compared with other taxa [88, 89].

There are several plausible explanations for the conserved response of the relative increases in lipid, fatty acid, and carbohydrate with increasing light transmittance, these include: (i) increased growth rate (assuming a pre-bloom phase), (ii) increased requirement for carbon storage in preparation for dormancy (assuming a post-bloom phase), or (iii) the onset of nutrient limitation as a result of increasing algal biomass restricting nutrient influx within the ice. It is likely that the higher light communities (HLT) were experiencing higher growth rates relative to the LLT communities, because of the greater light availability driving more photosynthesis [90]. The samples from the HLT sites were also more enriched with ^13^C, a parameter often associated with higher growth rates in microalgal species [91], lending further support to the idea that the increased lipid and carbohydrates were a result of higher growth rates. Whilst surrounding CO_2_ concentrations were not evaluated, they were unlikely to have influenced ^13^C enrichment, as the CO_2_ concentration in brine channels is typically determined by temperature, which was consistent across our sites [92]. Under the assumption that the ^13^C is because of higher growth rates, the increase in lipid content may be attributed primarily to structural lipids, as microalgae tend to accumulate structural (polar), over storage (neutral), lipids alongside an increase in growth rate, and as a response to early season light intensification [30]. An increase in growth rate and structural lipid content may explain the concurrent increase observed in other biomolecules, including carboxylated molecules [93] and unsaturated FAs, as PUFAs are found primarily in structural lipids [94].

An additional explanation for the increased lipid content with higher light transmittance lies with the fact that many sea-ice algal species have a dormancy strategy for overwintering, in which the cells increase their carbon reserves whilst reducing cellular metabolic activity and pigment content [95, 96]. This well-described strategy means that the increase in lipid, fatty acid, and carbohydrate measured in the cells from HLT sites in this study may reflect a response to increased light at the end of the growth season, when energy storage becomes a priority [97]. This physiological response may also explain the higher allocation to protein content relative to lipid and carbohydrate observed at LLT sites, as cell growth and division may have had lower priority at the HLT sites. The end-of-season response of reduced growth and increased allocation to storage molecules such as lipid, carbohydrate (specifically triacylglycerides and the polysaccharide storage polymer, glucan), and MUFA content has been observed previously in ice algal communities in response to increasing irradiance and decreasing nutrients characteristic of a post-bloom phase [80, 94, 98, 99]. Whilst nutrients were not limiting in the water under the ice at any of the sampling sites in this study, nor did we detect any decline over time [58], nutrient limitation within the boundary layer of the under-ice community cannot be ruled out, especially as cell densities increased. That said, the relatively low biomass accumulation at all sites, supported by the low chlorophyll *a* values [100, 101], in addition with the ice C:N ratios being close to Redfield (6.6) in all sites except VM-4 and TF-2, makes the onset of nutrient limitation within the ice community, and therefore the third possible explanation, less likely. It is possible, that instead of one specific driver underpinning the measured response, a blend or cascade of these processes was at play, as both SAFA and unsaturated FA increased and responses were often species-specific, as well as spatially and temporally diverse. Of note, we observed large lipid droplets within cells from HLT sites during microspectroscopic measurements (personal observation), indicating that lipid was being accumulated for storage in some cells. Such changes in biomolecular content have been observed in different organisms as an adaptation to seasonality in resource availability and life stage requirements [102]. Most importantly, however, whether driven by changes in energy allocation towards higher growth rates or increased energy storage in preparation for dormancy, the change in biomolecular content correlated with under-ice light environment, signifying the importance of light in determining food and essential nutrient availability to primary consumers.

The biomolecules more closely associated with functional cellular components were shown to vary with light transmissivity and across species, with some evidence of size-class grouping. Lipid (CH-stretch II) content increased with light in all taxa. Conversely, in all taxa, except *N. frigida*, we saw a decrease in phosphorylated molecules with increasing light. We saw a more nuanced response in protein across the five taxa, with four taxa showing no change in cellular protein content with light, whereas for one of the smallest taxa, *Navicula* spp.*,* protein content was negatively correlated with under-ice light availability (up to 15% incoming PAR). The lack of protein changes in the larger taxa corresponds with a previous study that showed protein content in sea-ice algae to remain stable, independent of light conditions [56]. In contrast to our findings, earlier work observed higher protein content under low light conditions [28, 103], but this was attributed to higher nutrients in the surrounding environment, rather than the low light conditions [104]. This finding may indicate a potential size-specific biomolecular response, in which smaller taxa have a reduced requirement to allocate energy into protein compared with larger taxa.

In addition, we found silica content declined with increasing light transmittance in all five taxa with the exception of an upturn in silica content at the highest light level in *N. frigida*. Similar trends in decline have been observed previously in light levels up to 150 μmol m^−2^ s^−1^ [105], whereas the opposite direction of change has been shown at particularly elevated light levels (300 μmol m^−2^ s^−1^) [106], meaning that there may be different mechanisms at play with respect to changes in diatom silicification and making the insights from this natural community study an important addition. The decrease in silica content observed with higher light conditions in this study could be attributed to higher growth rates. Changes in silicification has the potential to affect zooplankton grazing efficiency, as zooplankton have been shown to preferentially graze on less silicified diatoms [107] and reduced silica content may also mean the diatoms are more buoyant and therefore able to remain in the photic zone for longer when released from the sea ice, suggestive of potential changes to carbon flux.

The relationships between both photosynthetically derived and functional biomolecules and under-ice light have implications on energy supplied to the marine food web. In considering the prediction that parts of the Arctic may experience higher snowfall in the short-medium term [14, 23], and therefore less light under the sea ice, our results indicate that such environmental conditions would be concomitant with a shorter productive season, lower growth rates, and biomass accumulation, as well as a lower lipid, carbohydrate, fatty acid, and lipid (CH-stretch II) content in Arctic sea-ice algae. This would have significant implications for secondary production and beyond, with a reduced supply of organic carbon. In addition, critical biomolecules that are produced de novo, such as SAFAs, would likely be reduced, limiting supply to higher trophic levels [94]. Such reductions would be expected to have ramifications on secondary production and zooplankton fecundity [5]. Counter to the forecast of higher snowfall, if the increased precipitation comes in the form of rain or equally, as the warming ocean and air temperatures reduce ice thickness, then the under-ice light climate would increase. According to our data, the Arctic sea-ice algal communities could be expected to reduce silica content whilst increasing their lipid, carbohydrate, and fatty acid stores, at least until a certain threshold of incoming irradiance, beyond which could lead to photoinhibition, limiting photosynthetic energy production, and thereby biomolecular synthesis [81]. Higher under-ice light is likely to result in a higher relative abundance of *Navicula* spp., at the expense of the typically more dominant *N. frigida* [58], meaning that we might see even greater stores of lipid and carbohydrate with increasing under-ice light (beyond the threshold of 15% incoming PAR). Whilst higher under-ice light conditions may result in a community which is more calorific and nutrient rich, thinner sea ice conditions, and warmer ocean temperatures would likely shorten the ice-covered duration and/or result in earlier release of the community from the brine channels and therefore could result in a mismatch of energy supply for zooplankton reproduction [5]. In the most extreme case, where warming prevents sea ice from forming, the lack of substrate for sea-ice algae communities to develop would mean that this energy source would no longer be available to fuel polar marine food webs as they emerge from winter darkness.

Our study has revealed the importance of characterizing taxonomically resolved biochemical changes under varying environmental conditions. This is particularly pertinent for Arctic marine ecosystems where the effects of climate change are already occurring. Whilst uncertainty remains about the direction and magnitude of change to future under-ice light regimes, the results herein indicate that the nutritional content of key ice algae taxa will vary in response to shifts in under-ice light conditions which may result in a net loss of nutritional output. In combination with environmentally driven shifts in Arctic sea-ice microalgal community composition, season duration, and biomass accumulation, these changes will have implications for the quality and quantity of energy supplied to the polar marine food web.

## Supplementary Material

Duncan_et_al_Supplementary_Materials_Proofed_ycad010

## Data Availability

All data and processing scripts are available in the open repository Figshare. DOI: 10.6084/m9.figshare.24629718.
