# Supplementary material for: Biomolecular profiles of Arctic sea-ice diatoms highlight the role of under-ice light in cellular energy allocation
Source: ISME Commun. 2024 Jan 10;4(1):ycad010. doi: 10.1093/ismeco/ycad010 (PMC10848308; doi:10.1093/ismeco/ycad010)
Supplement: Duncan_et_al_Supplementary_Materials_Proofed_ycad010 [file duncan_et_al_supplementary_materials_proofed_ycad010.docx]

**Supplementary Information:**

**Title:** Biomolecular profiles of Arctic sea ice diatoms highlight the role of under-ice light in cellular energy allocation

**Authors**: Rebecca J. Duncan, Daniel Nielsen, Janne E. Søreide, Øystein Varpe, Mark J. Tobin, Vanessa Pitusi, Phillip Heraud, Katherina Petrou

**Table S1:** Location and dates of sampling events, with longitude and latitude provided in decimal degrees.

| **Location** | **Site** | **Longitude (N)** | **Latitude (E)** | **Sampling Date** |
| --- | --- | --- | --- | --- |
| Tempelfjorden | TF-2 | 78.43113 | 17.22808 | 30.4.21 |
| Van Mijenfjorden | VM-1 | 77.84918 | 16.7078 | 5.5.21 |
|  | VM-2 | 77.86545 | 16.70532 | 5.5.21 |
|  | VM-3 | 77.83154 | 16.30812 | 4.5.21 |
|  | VM-4 | 77.79405 | 15.8085 | 4.5.21 |
|  | VM-5 | 77.79996 | 15.7583 | 4.5.21 |

**Table S2:** Approximate cell length and width of the five taxa analysed

| **Taxa** | **Typical Length Range (μm)** | **Typical Width Range (μm)** | **Colonial/Solitary** | **Reference** |
| --- | --- | --- | --- | --- |
| *Entomoneis* spp. | 60-140 | 30-50 | Solitary | (Poulin & Cardinal, 1983) |
| *Haslea* spp. | 50-120 | 9-15 | Solitary | (Sterrenburg et al., 2015) |
| *Navicula* spp. | *N. Directa*:  72-105  *N. Transitas*:  50-105  *N. Valida*:  40-60 | *N. Directa*:  7-10  *N. Transitas*:  13-23  *N. Valida*:  15-25 | Solitary | (Poulin & Cardinal, 1982a)  (Tomas, 1997) |
| *Nitzschia frigida* | 45-75 | 5-7 | Colonial | (Medlin & Hasle, 1990) |
| *Pleurosigma* spp. | 100-300 | 15-30 | Solitary | (Poulin & Cardinal, 1982b) |

**Table S3:** Number of cells measured via synchrotron-based Fourier transform infrared microspectroscopy (s-FTIR) per site, per taxa

| **Site** | **Taxa** | **Cells Measured** |
| --- | --- | --- |
| VM-1 | *Entomoneis* spp*.* | 10 |
|  | *Haslea* spp. | 10 |
|  | *Navicula* spp. | 18 |
|  | *Nitzschia frigida* | 17 |
|  | *Pleurosigma* spp. | 7 |
| VM-2 | *Entomoneis* spp*.* | 11 |
|  | *Haslea* spp. | 12 |
|  | *Navicula* spp. | 20 |
|  | *Nitzschia frigida* | 17 |
|  | *Pleurosigma* spp. | 9 |
| VM-3 | *Entomoneis* spp*.* | 11 |
|  | *Haslea* spp. | 12 |
|  | *Navicula* spp. | 15 |
|  | *Nitzschia frigida* | 20 |
|  | *Pleurosigma* spp. | 12 |
| VM-4 | *Entomoneis* spp*.* | 14 |
|  | *Haslea* spp. | 9 |
|  | *Navicula* spp. | 20 |
|  | *Nitzschia frigida* | 20 |
|  | *Pleurosigma* spp. | 3 |
| VM-5 | *Entomoneis* spp*.* | 9 |
|  | *Haslea* spp. | 13 |
|  | *Navicula* spp. | 17 |
|  | *Nitzschia frigida* | 17 |
|  | *Pleurosigma* spp. | 9 |
| TF-2 | *Entomoneis* spp*.* | 14 |
|  | *Haslea* spp. | 1 |
|  | *Navicula* spp. | 17 |
|  | *Nitzschia frigida* | 19 |
|  | *Pleurosigma* spp. | 2 |

**Table S4:** Output of Spearman’s rank correlation coefficient (Multiple R^2^ and associated p-values) between environmental variables (% incoming photosynthetic active radiation (PAR), nitrite + nitrate (NO_3_^−^ + NO_2_^−^) (NO_x_) concentration in the water at ice-water interface, silicate (Si(OH)_4_) concentration in the water at ice-water interface, ice temperature (°C), under ice water temperature (°C) and bulk ice salinity (°C)) and biomolecular content (based on mean of each of the five taxa, per each set of pooled cores (total n = 86). Statistically significant p-values (< 0.05) and R^2^ exceeding 0.5 or -0.5 are marked in bold. Mean (x̄) ± standard deviation (σ) for each environmental variable is provided in brackets.

|  | **% Incoming PAR** | | **NO_3_**  (µM) | | **Si(OH)_4_**  (µM) | | **Ice Temperature (°C)** | | **Under-Ice Water Temperature**  **(°C)** | | **Bulk Ice Salinity** | |
| --- | --- | --- | --- | --- | --- | --- | --- | --- | --- | --- | --- | --- |
|  | (x̄ = 9.3, σ = ± 9.0) | | (x̄ = 2.1, σ = ± 0.1) | | (x̄ = 2.7, σ = ± 0.1) | | (x̄ = -2.3, σ = ± 0.2) | | (x̄ = -1.8, σ = ± 0.1) | | **(**x̄ = 7.7, σ = ± 2.8) | |
|  | **R^2^** | ***P*** | **R^2^** | ***P*** | **R^2^** | ***P*** | **R^2^** | ***P*** | **R^2^** | ***P*** | **R^2^** | ***P*** |
| Carbohydrate | -0.42 | **<0.01** | -0.36 | **<0.01** | 0.31 | **<0.05** | 0.23 | **0.03** | 0.03 | 0.78 | 0.11 | 0.30 |
| Carboxylated Molecules | -0.46 | **<0.01** | -0.34 | **<0.01** | 0.38 | **<0.05** | 0.28 | **0.01** | 0.01 | 0.92 | 0.17 | 0.11 |
| Lipid (Ester Carbonyl) | **0.81** | **<0.01** | 0.38 | **<0.01** | -0.21 | 0.05 | -0.45 | **<0.01** | 0.26 | **<0.01** | -0.37 | **<0.01** |
| Phosphorylated Molecules | -0.34 | **<<0.01** | -0.15 | 0.17 | 0.01 | 0.94 | 0.17 | 0.11 | -0.12 | 0.34 | 0.13 | 0.23 |
| Protein (Amide II) | -0.11 | 0.30 | -0.13 | 0.23 | 0.17 | 0.12 | 0.05 | 0.64 | -0.01 | 0.96 | 0.08 | 0.49 |
| Lipid (CH-stretch II) | **0.72** | **<0.01** | 0.34 | **<0.01** | -0.23 | **0.04** | -0.44 | **<0.01** | 0.15 | 0.22 | -0.27 | **0.01** |
| Saturated Fatty Acids | **0.82** | **<0.01** | 0.34 | **<0.01** | -0.19 | 0.08 | -0.48 | **<0.01** | 0.22 | 0.07 | -0.33 | **<0.01** |
| Saturated Lipid | **0.83** | **<0.01** | 0.37 | **<0.01** | -0.21 | 0.05 | -0.47 | **<0.01** | 0.24 | 0.05 | -0.35 | **<0.01** |
| Silica | **-0.52** | **<0.01** | -0.19 | 0.08 | 0.05 | 0.63 | 0.29 | **0.01** | -0.18 | 0.13 | 0.23 | **0.04** |
| Unsaturated fatty acids | **0.74** | **<0.01** | 0.28 | **0.01** | -0.19 | 0.08 | -0.49 | **<0.01** | 0.12 | 0.32 | -0.22 | **0.04** |

**Table S5:** Statistical output of the species-specific regression models. Multiple R^2^, adjusted R^2^, F statistic, associated degrees of freedom (DF) and p-value, Spearman’s rank correlation coefficient and associated p-value. Statistically significant p-values (< 0.05) are marked in bold.

|  |  | **Multiple R^2^** | **Adjusted R^2^** | **F Statistic** | **DF** | ***P* Value** | ***Spearman’s Correlation*** |  | ***P* Value (Spearman’s)** |
| --- | --- | --- | --- | --- | --- | --- | --- | --- | --- |
| ***Entomoneis* spp*.*** | Carbohydrate | 0.45 | 0.44 | 52.91 | 1,66 | **5.27 x 10^-10^** | 0.70 |  | **2.18 x 10^-11^** |
|  | Carboxylated Molecules | 0.21 | 0.19 | 17.11 | 1,66 | **1.02 x 10^-4^** | -0.44 |  | **1.49 x 10^-4^** |
|  | Lipid (CH-stretch II) | 0.44 | 0.43 | 52.37 | 1,66 | **6.14 x 10^-10^** | 0.71 |  | **1.09 x 10^-11^** |
|  | Lipid (Ester Carbonyl) | 0.52 | 0.51 | 71.53 | 1,66 | **4.02 x 10^-12^** | 0.78 |  | **6.75 x 10^-15^** |
|  | Phosphorylated Molecules | 0.28 | 0.27 | 26.23 | 1,66 | **2.83 x 10^-6^** | -0.62 |  | **1.24 x 10^-8^** |
|  | Protein (Amide II) | 0.12 | 0.11 | 9.35 | 1,66 | **0.01** | -0.41 |  | **4.46 x 10^-4^** |
|  | Saturated Fatty Acids | 0.51 | 0.50 | 68.08 | 1,66 | **9.44 x 10^-12^** | 0.74 |  | **6.73 x 10^-13^** |
|  | Saturated Lipid | 0.54 | 0.53 | 77.41 | 1,66 | **9.94 x 10^-13^** | 0.75 |  | **1.52 x 10^-13^** |
|  | Unsaturated Fatty Acids | 0.61 | 0.60 | 101.3 | 1,66 | **5.86 x 10^-15^** | 0.80 |  | **2.20 x 10^-16^** |
| ***Haslea* spp.** | Carbohydrate | 0.08 | 0.06 | 4.70 | 1,55 | **0.03** | 0.34 |  | **0.01** |
|  | Carboxylated Molecules | 0.25 | 0.24 | 18.48 | 1,55 | **7.08 x 10^-5^** | -0.44 |  | **5.97 x 10^-4^** |
|  | Lipid (CH-stretch II) | 0.27 | 0.25 | 20.09 | 1,55 | **3.80 x 10^-5^** | 0.52 |  | **3.12 x 10^-5^** |
|  | Lipid (Ester Carbonyl) | 0.46 | 0.45 | 46.49 | 1,55 | **7.48 x 10^-9^** | 0.64 |  | **8.18 x 10^-8^** |
|  | Phosphorylated Molecules | 0.12 | 0.10 | 7.53 | 1,55 | **8.18 x 10^-3^** | -0.37 |  | **0.005** |
|  | Protein (Amide II) | 0.00 | 0.00 | 0.54 | 1,55 | 0.47 | -0.09 |  | 0.52 |
|  | Saturated Fatty Acids | 0.39 | 0.38 | 35.67 | 1,55 | **1.78 x 10^-7^** | 0.60 |  | **8.92 x 10^-7^** |
|  | Saturated Lipid | 0.44 | 0.43 | 43.51 | 1,55 | **1.73 x 10^-8^** | 0.64 |  | **1.03 x 10^-7^** |
|  | Unsaturated Fatty Acids | 0.18 | 0.16 | 12.01 | 1,55 | **1.03 x 10^-3^** | 0.42 |  | **8.48 x 10^-4^** |
| ***Navicula* spp.** | Carbohydrate | 0.42 | 0.42 | 77.19 | 1,105 | **3.26 x 10^-14^** | 0.65 |  | **5.56 x 10^-14^** |
|  | Carboxylated Molecules | 0.28 | 0.28 | 41.74 | 1,105 | **3.32 x 10^-9^** | -0.49 |  | **1.17 x 10^-7^** |
|  | Lipid (CH-stretch II) | 0.46 | 0.46 | 90.31 | 1,105 | **7.80 x 10^-16^** | 0.65 |  | **2.08 x 10^-14^** |
|  | Lipid (Ester Carbonyl) | 0.53 | 0.53 | 119.6 | 1,105 | **2.2 x 10^-16^** | 0.74 |  | **2.20 x 10^-16^** |
|  | Phosphorylated Molecules | 0.21 | 0.20 | 27.82 | 1,105 | **7.20 x 10^-7^** | -0.55 |  | **9.37 x 10^-10^** |
|  | Protein (Amide II) | 0.14 | 0.13 | 16.67 | 1,105 | **8.69 x 10^-5^** | -0.35 |  | **2.68 x 10^-4^** |
|  | Saturated Fatty Acids | 0.52 | 0.51 | 109.6 | 1,105 | **2.20 x 10^-16^** | 0.72 |  | **2.20 x 10^-16^** |
|  | Saturated Lipid | 0.54 | 0.54 | 124.2 | 1,105 | **2.20 x 10^-16^** | 0.75 |  | **2.20 x 10^-16^** |
|  | Unsaturated Fatty Acids | 0.59 | 0.58 | 149.6 | 1,105 | **2.20 x 10^-16^** | 0.74 |  | **2.20 x 10^-16^** |
| ***Nitzschia frigida*** | Carbohydrate | 0.26 | 0.25 | 37.79 | 1,108 | **1.35 x 10^-8^** | 0.47 |  | **1.66 x 10^-7^** |
|  | Carboxylated Molecules | 0.26 | 0.26 | 38.88 | 1,108 | **8.95 x 10^-9^** | -0.43 |  | **2.71 x 10^-6^** |
|  | Lipid (CH-stretch II) | 0.63 | 0.62 | 182.4 | 1,108 | **2.20 x 10^-16^** | 0.76 |  | **2.20 x 10^-16^** |
|  | Lipid (Ester Carbonyl) | 0.68 | 0.68 | 230.6 | 1,108 | **2.20 x 10^-16^** | 0.82 |  | **2.20 x 10^-16^** |
|  | Phosphorylated Molecules | 0.02 | 0.00 | 2.07 | 1,108 | 0.15 | 0.07 |  | 0.44 |
|  | Protein (Amide II) | 0.00 | 0.00 | 0.27 | 1,108 | 0.61 | 0.08 |  | 0.41 |
|  | Saturated Fatty Acids | 0.72 | 0.72 | 278.0 | 1,108 | **2.20 x 10^-16^** | 0.80 |  | **2.20 x 10^-16^** |
|  | Saturated Lipid | 0.72 | 0.72 | 280.6 | 1,108 | **2.20 x 10^-16^** | 0.82 |  | **2.20 x 10^-16^** |
|  | Unsaturated Fatty Acids | 0.40 | 0.38 | 70.49 | 1,108 | **1.98 x 10^-13^** | 0.65 |  | **2.47 x 10^-14^** |
| ***Pleurosigma* spp.** | Carbohydrate | 0.28 | 0.26 | 15.52 | 1,40 | **3.19 x 10^-4^** | 0.47 |  | **0.002** |
|  | Carboxylated Molecules | 0.18 | 0.16 | 9.04 | 1,40 | **0.005** | -0.30 |  | 0.06 |
|  | Lipid (CH-stretch II) | 0.36 | 0.33 | 21.62 | 1,40 | **3.59 x 10^-5^** | 0.60 |  | **2.79 x 10^-5^** |
|  | Lipid (Ester Carbonyl) | 0.48 | 0.46 | 36.21 | 1,40 | **2.47 x 10^-7^** | 0.75 |  | **1.04 x 10^-8^** |
|  | Phosphorylated Molecules | 0.17 | 0.15 | 8.17 | 1,40 | **0.007** | -0.47 |  | **0.001** |
|  | Protein (Amide II) | 0.06 | 0.04 | 2.6 | 1,40 | 0.11 | -0.07 |  | 0.63 |
|  | Saturated Fatty Acids | 0.48 | 0.46 | 36.58 | 1,40 | **4.04 x 10^-7^** | 0.69 |  | **2.62 x 10^-7^** |
|  | Saturated Lipid | 0.54 | 0.53 | 46.36 | 1,40 | **3.46 x 10^-8^** | 0.73 |  | **2.63 x 10^-8^** |
|  | Unsaturated Fatty Acids | 0.11 | 0.09 | 5.06 | 1,40 | **0.03** | 0.39 |  | **0.01** |

**Table S6:** Statistical output of the biomolecular regression models. Multiple R^2^, adjusted R^2^, F statistic, associated degrees of freedom (DF) and p-value. Statistically significant p-values (< 0.05) are marked in bold. The low light transmissivity sites include VM-1, VM-2 and VM-3 and the high light transmissivity sites include VM-4, VM-5 and TF-2.

|  |  | **Multiple R^2^** | **Adjusted R^2^** | **F Statistic** | **DF** | ***P* Value** |
| --- | --- | --- | --- | --- | --- | --- |
| **Lipid vs. Protein**  ***Low Light*** | *Entomoneis* spp*.* | 0.44 | 0.42 | 22.48 | 1,29 | **5.21 x 10^-5^** |
|  | *Haslea* spp. | 0.66 | 0.65 | 62.34 | 1,32 | **5.21 x 10^-9^** |
|  | *Navicula* spp. | 0.34 | 0.33 | 26.5 | 1,51 | **4.27 x 10^-6^** |
|  | *Nitzschia frigida* | 0.35 | 0.34 | 28.55 | 1,52 | **2.05 x 10^-6^** |
|  | *Pleurosigma* spp. | 0.62 | 0.61 | 41.76 | 1,26 | **7.56 x 10^-7^** |
| **Lipid vs. Protein**  ***High Light*** | *Entomoneis* spp*.* | 0.20 | 0.18 | 9.00 | 1,35 | **0.005** |
|  | *Haslea* spp. | 0.62 | 0.60 | 34.59 | 1,21 | **7.73 x 10^-6^** |
|  | *Navicula* spp. | 0.01 | 0.00 | 0.61 | 1,52 | 0.44 |
|  | *Nitzschia frigida* | 0.00 | 0.00 | 0.30 | 1,54 | 0.58 |
|  | *Pleurosigma* spp. | 0.03 | 0.00 | 0.38 | 1,12 | 0.55 |
| **Lipid vs. Carbohydrate**  ***Low Light*** | *Entomoneis* spp*.* | 0.15 | 0.12 | 5.23 | 1,29 | **0.03** |
|  | *Haslea* spp. | 0.00 | 0.00 | 0.01 | 1,32 | 0.91 |
|  | *Navicula* spp. | 0.08 | 0.06 | 4.44 | 1,51 | **0.04** |
|  | *Nitzschia frigida* | 0.03 | 0.01 | 1.67 | 1,52 | 0.21 |
|  | *Pleurosigma* spp. | 0.00 | 0.00 | 0.10 | 1,26 | 0.75 |
| **Lipid vs. Carbohydrate**  ***High Light*** | *Entomoneis* spp*.* | 0.62 | 0.61 | 57.84 | 1,35 | **6.34 x 10^-9^** |
|  | *Haslea* spp. | 0.57 | 0.55 | 28.29 | 1,21 | **2.83 x 10^-5^** |
|  | *Navicula* spp. | 0.35 | 0.33 | 27.81 | 1,52 | **2.63 x 10^-6^** |
|  | *Nitzschia frigida* | 0.17 | 0.16 | 11.19 | 1,54 | **0.001** |
|  | *Pleurosigma* spp. | 0.87 | 0.86 | 83.18 | 1,12 | **9.56 x 10^-7^** |
| **Carbohydrate vs. Protein**  ***Low Light*** | *Entomoneis* spp*.* | 0.00 | 0.00 | 0.07 | 1,29 | 0.79 |
|  | *Haslea* spp. | 0.04 | 0.01 | 1.34 | 1,32 | 0.26 |
|  | *Navicula* spp. | 0.00 | 0.00 | 0.24 | 1,51 | 0.63 |
|  | *Nitzschia frigida* | 0.06 | 0.04 | 3.10 | 1,52 | 0.08 |
|  | *Pleurosigma* spp. | 0.00 | 0.00 | 0.01 | 1,26 | **0.90** |
| **Carbohydrate vs. Protein**  ***High Light*** | *Entomoneis* spp*.* | 0.11 | 0.09 | 4.36 | 1,35 | **0.04** |
|  | *Haslea* spp. | 0.40 | 0.36 | 13.58 | 1,21 | **0.001** |
|  | *Navicula* spp. | 0.00 | 0.00 | 0.00 | 1,52 | 0.94 |
|  | *Nitzschia frigida* | 0.03 | 0.00 | 1.40 | 1,54 | 0.24 |
|  | *Pleurosigma* spp. | 0.00 | 0.00 | 0.05 | 1,12 | 0.82 |

**Figure S1:**


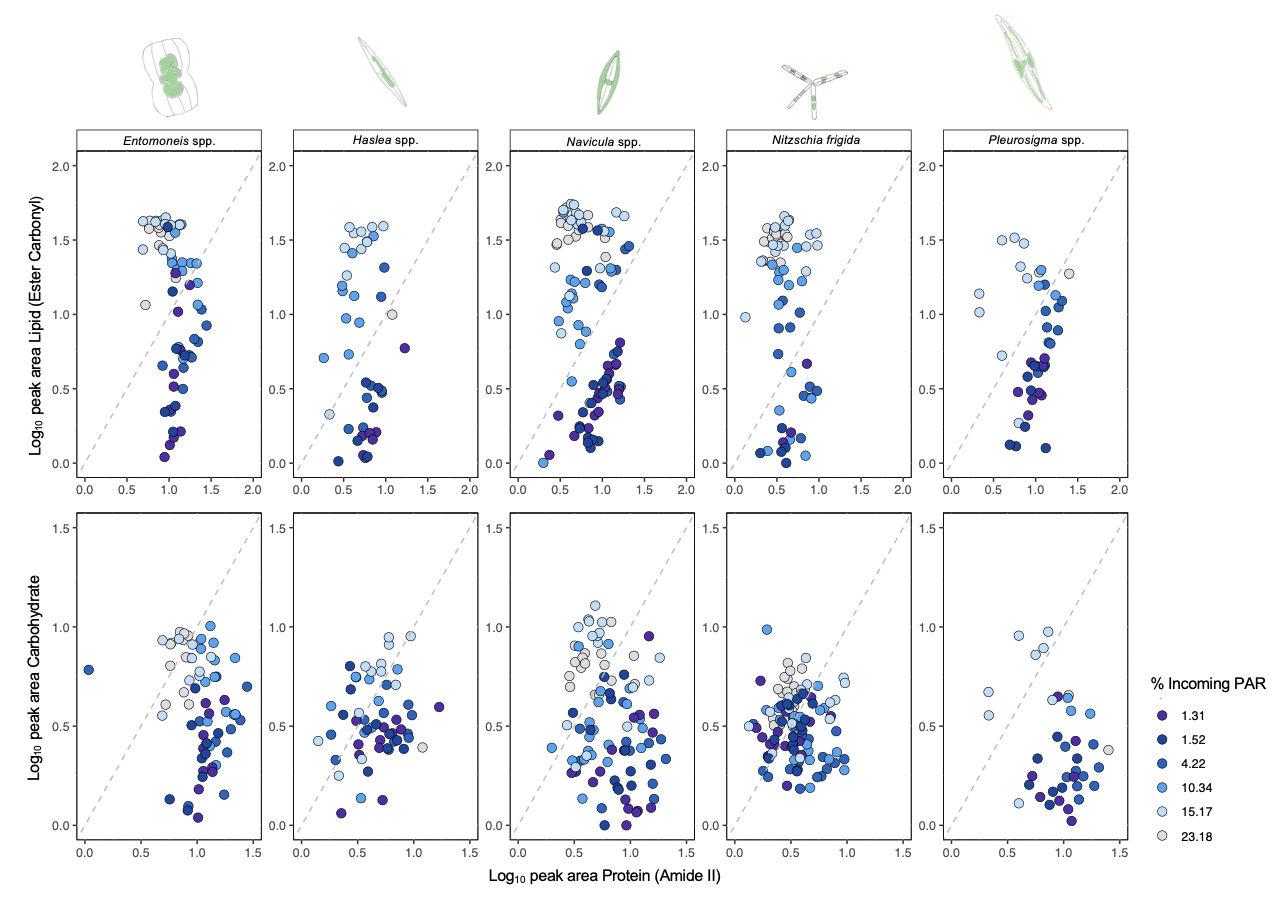


**References:**

Giordano, M., Kansiz, M., Heraud, P., Beardall, J., Wood, B., & McNaughton, D. (2001). Fourier transform infrared spectroscopy as a novel tool to investigate changes in intracellular macromolecular pools in the marine microalga Chaetoceros muellerii (Bacillariophyceae). *Journal of Phycology*, *37*(2), 271-279.

Medlin, L. K., & Hasle, G. R. (1990). Some Nitzschia and related diatom species from fast ice samples in the Arctic and Antarctic. *Polar Biology*, *10*, 451-479.

Murdock, J. N., & Wetzel, D. L. (2009). FT-IR microspectroscopy enhances biological and ecological analysis of algae. *Applied Spectroscopy Reviews*, *44*(4), 335-361.

Petrou, K., Nielsen, D. A., & Heraud, P. (2018). Single-cell biomolecular analysis of coral algal symbionts reveals opposing metabolic responses to heat stress and expulsion. *Frontiers in Marine Science*, *5*, 110.

Poulin, M., & Cardinal, A. (1982a). Sea ice diatoms from Manitounuk Sound, southeastern Hudson Bay (Quebec, Canada).: II. Naviculaceae, genus Navicula. *Canadian Journal of Botany*, *60*(12), 2825-2845.

Poulin, M., & Cardinal, A. (1982b). Sea ice diatoms from Manitounuk Sound, southeastern Hudson Bay (Quebec, Canada). I. Family Naviculaceae. *Canadian Journal of Botany*, *60*(7), 1263-1278.

Poulin, M., & Cardinal, A. (1983). Sea ice diatoms from Manitounuk Sound, southeastern Hudson Bay (Quebec, Canada). III. Cymbellaceae, Entomoneidaceae, Gomphonemataceae, and Nitzschiaceae. *Canadian Journal of Botany*, *61*(1), 107-118.

Sterrenburg, F. A., Tiffany, M. A., Hinz, F., Herwig, W. E., & Hargraves, P. E. (2015). Seven new species expand the morphological spectrum of Haslea. A comparison with Gyrosigma and Pleurosigma (Bacillariophyta). *Phytotaxa*, *207*(2), 143-162.

Tomas, C. R. (1997). *Identifying marine phytoplankton*. California, USA: Elsevier.

Vongsvivut, J., Heraud, P., Zhang, W., Kralovec, J. A., McNaughton, D., & Barrow, C. J. (2012). Quantitative determination of fatty acid compositions in micro-encapsulated fish-oil supplements using Fourier transform infrared (FTIR) spectroscopy. *Food chemistry*, *135*(2), 603-609.
